# Developing Interpreting Competence Scales in China

**DOI:** 10.3389/fpsyg.2020.00481

**Published:** 2020-04-23

**Authors:** Weiwei Wang, Yi Xu, Binhua Wang, Lei Mu

**Affiliations:** ^1^School of Interpreting and Translation Studies, Guangdong University of Foreign Studies, Guangzhou, China; ^2^Centre for Translation Studies, University of Leeds, Leeds, United Kingdom

**Keywords:** interpreting competence, assessment and scales, descriptors, China standards of english, scale development and validation

## Abstract

Tertiary-level interpreter training and education have developed rapidly in China, and over 200 undergraduate and over 200 postgraduate T&I programs have been launched over the past decade. Despite the rapid development, there has been no standardized framework allowing for the reliable and valid measurement of interpreting competence in China. Against this background, the China Standards of English (CSE), which are the Chinese counterpart to the Common European Framework of Reference (CEFR), were unveiled in 2018 after 4 years of government-funded research and validation. One vital component of the CSE is the descriptor-referenced interpreting competence scales. This article provides a systematic account of the design, development, and validation of the interpreting competence scales in China. Within the CSE, the construct of interpreting competence was defined according to an interactionist approach. It not only encompasses cognitive abilities, interpreting strategies, and subject-matter knowledge but also considers performance in typical communicative settings. Based on the construct definition, a corpus of relevant descriptors was built from three main sources, including: (a) interpreting training syllabuses, curricular frameworks, rating scales, and professional codes of conduct; (b) previous literature on interpreting performance assessment, competence development, and interpreter training and education; and (c) exemplar-generation data on assessing interpreting competence and typical interpreting activities, which were collected from interpreting professionals, trainers, and trainees. The corpus contains 9,208 descriptors of interpreting competence. A mixed-method survey was then conducted to analyze, scale, and validate the descriptors among 30,682 students, 5,787 teachers, and 139 interpreting professionals from 28 provinces, municipalities, and regions in China. The finalized set included 369 descriptors that reference interpreting competence. The CSE—Interpreting Competence Scales with theoretically and empirically based descriptors represent a major effort in research on interpreting competence and its assessment, and they have significant potential to be applied widely in interpreting training, research, and assessment.

## Introduction

In the mid-twentieth century, universities began to offer programs designed to train conference interpreters (Pöchhacker, 2015), and the first group of programs was offered in Moscow (1930), Heidelberg (1933), Geneva (1941), and Vienna (1943). Since then, more universities have developed interpreter education programs. As of 2016, the International Association of Conference Interpreters (AIIC) had listed 95 programs in its Interpreting Schools and Programs Directory. By 2019, the European Masters in Conference Interpreting (EMCI) had endorsed 16 programs through their member universities. Over the past decade, China has witnessed a rapid growth in translator and interpreter education. By March 2019, over 282 Chinese universities had Bachelor’s degree programs in Translation and Interpreting (BTI), and 249 Chinese universities had Master’s degree programs in Translation and Interpreting (MTI).

Chinese interpreting training programs differ from their European counterparts in a number of ways. First, Translation and Interpreting (T&I) programs in China offer both undergraduate and postgraduate training, while most European T&I programs offer postgraduate training. Second, Chinese T&I students need to work bi-directionally, including retour into their B languages (i.e. second language); this has long been a professional norm in the Chinese interpreting market. Western interpreters, meanwhile, often interpret into their A, or first, language (Seleskovitch and Lederer, 1989). Third, although students enrolled in T&I programs are expected to have a high level of general and cultural knowledge and adequate B language proficiency, i.e. Interagency Language Roundtable (ILR) Band 3 or 4 or Common European Framework of Reference for Languages (CEFR) C1 or C2 (Setton and Dawrant, 2016), experience in China shows that most students still need B language enhancement. The reason behind this deficit is that most students in China learn English through formal classroom teaching and are often deficient in conversational listening and speaking due to the limited opportunities for immersive English language learning with native speakers. In comparison, European T&I students tend to have higher B and C language (third language) proficiency (Szabari, 2002).

With over 200 undergraduate and over 200 postgraduate T&I degree programs being launched over the past decade in China, the lack of consistent teaching practices and common competence standards has become an urgent issue in the training and assessment of interpreters. To address the issue, interpreting educators and researchers have worked collaboratively on a national-level project led by the Ministry of Education: the China Standards of English (CSE). The purpose of CSE was to develop a national framework of interpreting competence that could support T&I students’ professional development, and scales of interpreting competence to provide guidance for interpreting training and assessment in China.

Previous research on interpreting competence has focused on different areas, “most notably cognitive processes, education (including curriculum design, aptitude testing, and pedagogy) and certification programs” (Pöchhacker, 2015, 69). Subsequently, very few models or frameworks for interpreting competence have been put forward. Most of the available models examined the composition of interpreting competence. For instance, Pöchhacker (2000) proposed a multidimensional model of interpreting competence that highlighted language and cultural skills, translational skills, and subject-matter knowledge. In this model, linguistic transfer competence was regarded as a core element, complemented by cultural competence and interaction management skills. These elements were all supported by professional performance skills and ethical behavior (Pöchhacker, 2015). Albl-Mikasa (2013) referred to interpreting models suggested by Kalina (2002) and Kutz (2010) when proposing a detailed model that comprises five skill sets, each with a set of sub-skills: pre-process (language proficiency, terminology management, and preparation); in-process (comprehension, transfer, and production); peri-process (teamwork and ability to handle stress); post-process (terminology work and quality control); and para-process (business acumen, customer relations, and meta-reflection). Han (2015) applied an interactionist approach to construct the components of interpreting ability, including knowledge of languages, interpreting strategies, topical knowledge, and metacognitive process. Dong (2018) researched the development of students’ interpreting competence through longitudinal empirical data and proposed a complex dynamic system to illustrate how self-organization among different key parameters results in interpreting competence.

When defining interpreting competence, bilingual linguistic competence and professionalism are frequently mentioned by scholars. For instance, Kalina (2000) defined interpreting competence from a psycholinguistic perspective, calling it the ability to process texts in a bilingual or multilingual communication environment. Zhong (2003) proposed that interpreting competence should include linguistic knowledge, encyclopedic knowledge, and skills related to both professional interpreting and artistic presentation. Wang (2007, 2012) defined interpreting competence as the underlying system of knowledge and skills required to accomplish the task of interpreting, including the necessary professional and physio-psychological qualities. Setton and Dawrant (2016) stated that interpreting competence is composed of four core elements: bilingual language proficiency, knowledge, skills, and professionalism.

The models or definitions of interpreting competence mentioned above indicate that researchers agree that interpreting competence goes beyond simple bilingual competence and includes skills of cross-cultural communication. They also demonstrate that, although there is no universally accepted model of interpreting competence, the previous discussions illustrate the composition of interpreting competence. It is also clear that little attention has been paid to the developmental stages of interpreting competence and that the different competence requirements for specific interpreting tasks have been overlooked.

When assessing interpreting competence, research in interpreting quality is highly relevant. The literature on the concept of interpreting quality, assessment, and evaluation is extensive (e.g. Barik, 1975; Berk-Seligson, 1988; Kurz, 1993; Moser-Mercer, 1996; Aís, 1998; Campbell and Hale, 2003; Napier, 2004a, b; Kalina, 2005a, b; Liu, 2008; Gile, 2011). For instance, Liu (2008) studied the differences in interpreting competence by comparing the performance of expert and novice interpreters. Research on certification also provides effective instruments and outlines potential problems for assessing interpreting competence (Pym et al., 2013). In terms of quality parameters, many scholars have proposed criteria including completeness, accuracy, intonation, voice projection, language use, and logical cohesion (Kurz, 1993; Moser-Mercer, 1996; Garzone, 2003). Pöchhacker (2001) suggested four common criteria to cover the quality range from product to service: accurate rendition, adequate target language expression, equivalent intended effect, and successful communicative interaction. As Pöchhacker (2015, p.334) put it, “on a superficial level, quality relates to something that is good or useful, or to behavior that is sanctioned or expected.” However, it is difficult to measure interpreting quality quantitatively given its complexity. Grbić (2008), in her conceptual study of interpreting quality, proposed that interpreting quality should be assessed based on actual settings. The past decades witnessed a distinct strand of research on Interpreting Quality Assessment (IQA) especially in the educational context (Yeh and Liu, 2006; Lee, 2008; Postigo Pinazo, 2008; Tiselius, 2009; Liu, 2013; Lee, 2015; Wang et al., 2015; Han, 2016, 2017, 2018, 2019; Han and Riazi, 2018; Lee, 2018, 2019). Among them, the design and application of rating scales that assess interpreting quality have been a priority for many interpreting researchers and trainers. Based on the prior literature, interpreting quality constructs and parameters have been operationalized into various rubric-referenced rating scales. For instance, Liu (2013) discussed the development of the rating scheme for Taiwan’s interpretation certification exam. Lee (2015) has provided a detailed report on the process of developing an analytic rating scale for assessing undergraduate students’ consecutive interpreting performances. Han (2017, 2018) probed into the application and validity of rating scales for students’ English–Chinese consecutive interpreting performance. Despite the popularity of assessment rubrics and rating scales, competence-based scales that could describe the progressive development of interpreters at different levels and diagnose skills gaps have not been developed.

While research in IQA has been a prominent topic in interpreting studies, the construct and measurement of the progressive stages of interpreting competence have received limited attention. The ILR, as the earliest language proficiency scale developed by the United States government in 1955, is the only assessment scale that includes interpreting. ILR describes interpreting performance in three bands: Professional Performance (Levels 3 to 5), Limited Performance (Levels 2 and 2+), and Minimal Performance (Levels 1 and 1+). In ILR, only individuals performing at the Professional Performance levels are properly termed “interpreters” (Interagency Language Roundtable, 2002). Since then, several language proficiency scales have been proposed in Europe, Canada, Australia, and other countries and regions. These include the American Council on the Teaching of Foreign Languages (2019) Proficiency Guidelines, the International Second Language Proficiency Ratings (ISLPR), and the Canadian Language Benchmarks (CLB) (Centre for Canadian Language Benchmarks, 2019). The CEFR scale, jointly developed by more than 40 members of the Council of Europe, is widely used in countries around the world (Council of Europe, 2001). However, none of these frameworks seem to focus on interpreting or have accounted for students’ progression from novice interpreter to expert interpreter.

Furthermore, understanding and describing the development of interpreting competence is even more pertinent due to the current challenges in education quality faced by the T&I degree programs in China. To this end, the Chinese Ministry of Education initiated the CSE Project to develop a national framework and a set of standards for Chinese–English interpreting students. This national project was supervised by the National Education Examinations Authority (National Education Examinations Authority, 2014).

In general, the CSE–Interpreting Competence Scales were developed with two broad aims: first, they were to act as a stimulus for reflection on current practice in the country; second, they were to provide a common reference for developing teaching syllabuses, curriculum guidelines, examinations, and textbooks for interpreting across China. The CSE–Interpreting Competence Scales were designed to contribute to educational reform and innovation in order to improve the efficiency of the teaching, learning, and assessment of interpreting.

**FIGURE 1:**
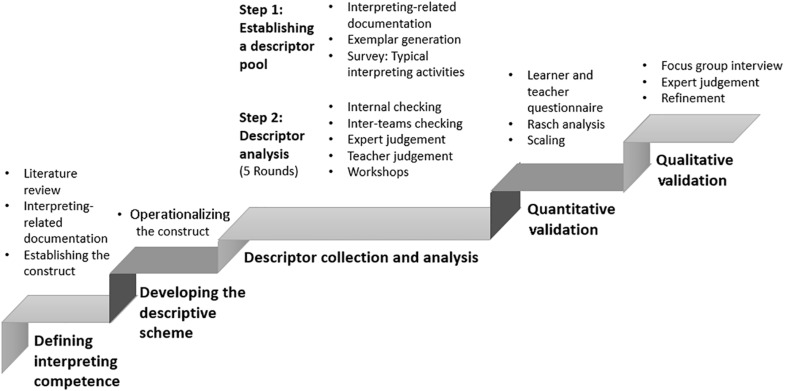
The development process of the China Standards of English (CSE)—Interpreting Competence Scales.

This paper reports on the development process of the CSE–Interpreting Competence Scales, which involved research work in two major parts, divided into five stages, as follows (Figure 1):

Part I: Drafting the scales: the creation of a descriptor pool.
Stage 1: Defining interpreting competence with respect to the Chinese–English interpreting training context of China
Stage 2: Developing an interpreting competence descriptive scheme
Stage 3: Collecting descriptors with reasonable representativeness
Part II: Validating the scales: scaling and refinement
Stage 1: Quantitative validation: main data collection and scaling descriptors through teacher and student assessments
Stage 2: Qualitative validation: consultation with teachers through focus group interviews and workshops

The remainder of this article is divided into four sections. The following two sections introduce the two stages that developed the CSE–Interpreting Competence Scales. The fourth section discusses the limitations and issues encountered in the design of the scales. Finally, the fifth section concludes with the possible application of the undertaken work.

## Drafting the Scale: Creation of a Descriptor Pool

The CSE–Interpreting Competence Scales cover four aspects of descriptors: overall interpreting performance, typical interpreting activities, interpreting strategies, and self-assessment scales. In this section, we will illustrate the process of drafting the interpreting competence scales. This development process is divided into three stages: defining interpreting competence, developing the descriptive scheme, and collecting descriptors.

### Defining Interpreting Competence

To fully account for the perceptions of different stakeholders (interpreting learners, trainers, testers, users, employers, policymakers, etc.), we constructed interpreting competence based on previous literature in interpreting studies and on Bachman’s (1990) communicative language competence model. Interpreting competence is demonstrated as decisions made by the interpreter to purposefully perform an interpreting task in a given place at a given time. The competence involves cognitive processing, language proficiency, extra-linguistic knowledge, and interpreting strategies.

Drawing on existing literature, we define interpreting competence as the interlingual and intercultural mediation ability of instantaneously transferring utterances from a source language into a target language, using language proficiency, related world knowledge, and interpreting-specific strategies.

**FIGURE 2:**
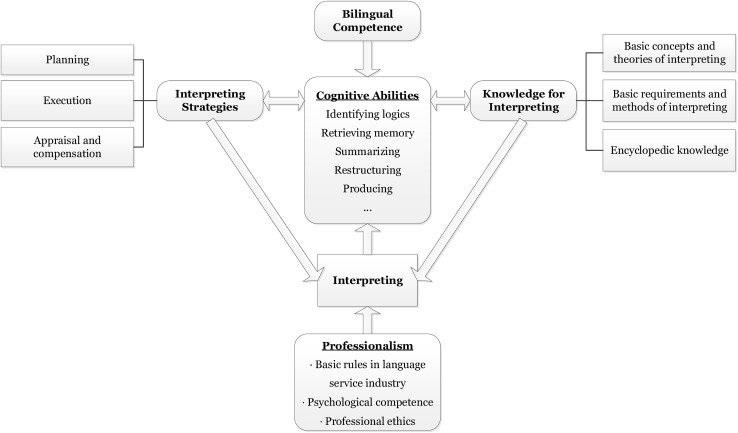
Construct of interpreting competence.

According to this definition, interpreting competence is, first and foremost, a comprehensive cognitive ability used in interpreting activities. It involves mechanisms and procedures of information processing (Bachman and Palmer, 1996). These activities include identifying the logic of the source text, retrieving memory, summarizing, and analyzing the structure of the source text. As Angelelli and Degueldre (2002) and many other scholars in interpreting studies have observed, bilingual proficiency is the prerequisite for interpreting. In this integrated process, the activity’s basis is bilingual competence in Chinese and English. The interpreter’s topic-specific and/or world knowledge plays a key role in the process of comprehension (Will, 2007; Díaz-Galaz, 2011; Díaz-Galaz et al., 2015; Fantinuoli, 2017). At the same time, interpreting strategies are used in both comprehension and reproduction (Kohn and Kalina, 1996; Bartłomiejczyk, 2006; Li, 2013, 2015; Arumí Ribas and Vargas-Urpi, 2017; Wu and Liao, 2018; Dong et al., 2019). The definition also includes professionalism, meaning that interpreters must abide by the code of conduct of the industry. They must be mentally ready to work under stress and make operational and ethical decisions aimed at optimizing interpretation in real life (Setton and Dawrant, 2016). The construct of interpreting competence is illustrated in Figure 2. As the cognitive process is invisible, our descriptions based on this construct focus on interpreting activities (i.e. interpreting modes, topics, and context) and products (performance).

### Development of the Descriptive Scheme

The scheme of the description serves to create a link between real-life tasks and the construct of interpreting competence. The “can-do” principle of CEFR (North, 2014) suggests that descriptors of interpreting competence scales typically consist of three elements:

(1) Performance: the interpreting task (e.g. “*interpreting a speech consecutively”*)
(2) Criteria: the intrinsic characteristics of the performance, involving a range of cognitive efforts or interpreting skills (e.g. “*actively anticipating speech information, with note-taking*”)
(3) Conditions: any extrinsic constraint or condition defining the performance (e.g. “*moderate speech rate, high information density, and with no accent*”)

The can-do principle describes the expected type of interpreting competence descriptors. The scheme of description determines the interpreting competence scale structure and reflects the interpreting competence construct defined above.

**FIGURE 3:**
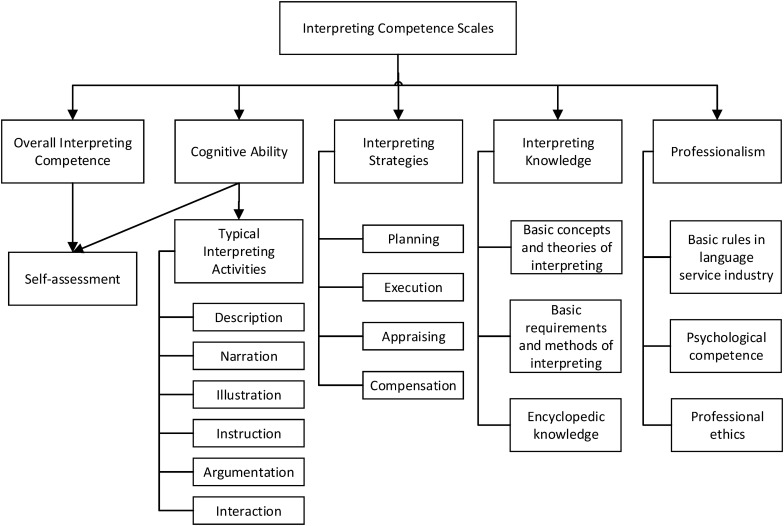
Operational descriptive scheme of the Interpreting Competence Scales.

Figure 3 illustrates an operational descriptive scheme that covers overall interpreting performance and cognitive ability, interpreting strategies, knowledge, and professionalism; this is a practical application of the theoretical presentation in Figure 2. Interpreting-related cognitive ability, interpreting strategies, and subject-matter knowledge are identified in the construct as the three core elements of interpreting competence. It must be noted that bilingual competence is not included in the scheme, because the listening, speaking, writing, and reading scales of the CSE have already covered descriptions of English language proficiency.

Cognitive abilities are the key components of the interpreting competence construct and are described via interpreting tasks in both the scales of overall interpreting performance and sub-sets of typical interpreting activities. One of the cognitive abilities, for instance, is described as “*Can understand the content of an interview while analyzing the logical relationships in the source-language information during SI for a media interview.*”

As evident in Figure 3, interpreting strategies are described in different subscales, i.e. planning, execution, appraisal, and compensation. The strategy scales refer mainly to the skills, methods, and actions that aim to fulfill interpreting tasks and solve problems. For instance, “*Can use contextual information to anticipate upcoming content and information actively.*”

Typical interpreting activities include business negotiations, training, lectures, interviews, and conferences. For instance, “*Can interpret important information, such as research objectives, methodology, and conclusions, during SI for an academic talk*” and “*Can follow target-language norms to reflect source-language register and style during Consecutive Interpreting (CI) with note-taking for a foreign affairs meeting.*” The knowledge scales include descriptors about encyclopedic knowledge, basic methods, theories of interpreting, and so on.

### Descriptor Collection and Analysis

Drawing on the theoretical concepts of interpreting competence and the operational descriptive scheme, we established a pool of interpreting descriptors based on documentation, exemplar generation, and surveys.

#### Step 1: Establishing a Descriptor Pool

##### Documentation: the analysis and editing of existing descriptions in interpreting training and research as well as the interpreting profession

Through documentation, we collected a wide range of materials such as existing scales related to interpreting (e.g. ILR); teaching syllabuses; curricular frameworks; textbooks; rating scales from the established interpreting programs such as the University of Leeds, Middlebury Institute of International Studies at Monterey, Shanghai International Studies University, and Guangdong University of Foreign Studies; test specifications and codes of conduct from established professional associations such as the AIIC and accreditation agencies such as the National Accreditation Authority for Translators and Interpreters (NAATI) in Australia and the China Accreditation Test for Translators and Interpreters (CATTI); and previous research on interpreting performance assessment, competence development, and interpreter training and education.

Due to the large number of interpreting-related documents, more than 110 postgraduate students in T&I majors were recruited as volunteers to help with the highly labor-intensive sorting and editing work. One of the authors of the article led training with the volunteers and then worked with them to collect 8,937 descriptors from 1,048 documents.

##### Exemplar generation: writing new descriptors from video performance of selective learners

For the exemplar generation, 22 videos of interpreting performances by students at different levels were recorded; 69 professionals and trainers were invited to write descriptors based on the videos. After rigorous training by the authors on the descriptive scheme and according to the three-element can-do principle, 16 trainers were asked to describe the actual interpreting competence of their students in terms of cognitive abilities, interpreting strategies, and knowledge. In total, 271 descriptors were generated during this process.

##### Survey: collecting descriptors for typical interpreting tasks

We surveyed 53 professional interpreters and 150 student interpreters with online questionnaires to collect their opinions about typical interpreting tasks. We also surveyed and examined relevant textbooks used for different stages of interpreter training and T&I industry reports, including the annual reports of the language service industry in China by the Translators Association of China (TAC).

In this step, a corpus containing a total of 9,208 potential descriptors of interpreting competence was established. Each of the descriptors was assigned into relevant levels and categories of the descriptive scheme.

#### Step 2: Analyzing Descriptors

In this step, we carried out several rounds of analysis to remove the redundant and overlapping descriptors, reformulate the ambiguous ones, and rewrite those inconsistent with the style and quality requirement for descriptors. Moreover, a glossary of verbs and nouns frequently used in the three categories (cognitive ability, strategies, and knowledge) was generated from the corpus to ensure terminology consistency.

##### The first round

Four groups composed of members from the interpreting scales’ author team checked the descriptors collected from different sources for redundancy and repetition. For instance, “accurate rendition,” “accurate delivery,” and “accuracy in interpretation” could be integrated. This round of sorting tasks consisted of an interactive process involving repetition checks in each set of descriptive categories; the redundancy analysis of each descriptor; and a series of workshops within each group to ensure the appropriate assignment of descriptors into relevant levels. The initial width of the interpreting scale was discussed within the author team. Level 6, which is the upper intermediate level of the nine levels of the CSE Project (Liu, 2019), was determined to be the beginner’s level of interpreting scales for two reasons. First, bilingual proficiency is the basis for interpreting. Second, interpreting courses start in the third semester of the BTI programs and the fourth or fifth semester for English majors in China. These student groups fell into the provisional Level 6 of CSE (Liu, 2019). As a result of the refinement, 3,259 descriptors remained.

##### The second round

Cross-group checking within the interpreting scales’ author team was undertaken to review the quality of the descriptors in this round. A similar task to check repetition, redundancy, wording, and classification was conducted. For instance, the descriptor “*Can process the information in the source speech to find the logic structure, such as the main idea, supporting idea and details*” was too long and involved unnecessary examples. It was therefore revised into “*Can identify the logical threads of the source speech.*” At this point, each descriptor was reviewed by at least three team members. A total of 1,081 descriptors survived this round of scrutiny.

##### The third round

The remaining descriptors were cross-checked further by researchers in the translation and the speaking scales team of the CSE Project. Two external experts in interpreting education and research were also invited to identify ambiguities or inappropriate expressions. In this round, the provisional levels of the descriptors were based on the judgments made by these experts and were based on their in-depth understanding of interpreting theories and practices. The issue of whether or not sight interpreting/translation should be regarded as a distinct interpreting working mode was discussed. Considering that sight interpreting/translation and its variants were mostly used at earlier stages of interpreting training, as a deverbalization exercise, or as preparatory training for simultaneous interpreting (Setton and Dawrant, 2016), we deleted the descriptors on sight interpreting/translation. By the end of this round, we managed to further reduce the number of descriptors to about 700.

##### The fourth round

Over 5,000 descriptors that had survived the first three rounds of analysis from all teams of the CSE Project (listening, speaking, reading, writing, translation, interpreting, etc.) were used to construct over 100 online questionnaires of 50 to70 items (i.e. descriptors) each. All the researchers in the CSE Project and over 50 external experts took part in this online reviewing process to provide feedback on wording (e.g. explicitness, clarity, and appropriateness), descriptor structure (e.g. performance, criteria, and condition), and provisional levels and classification (e.g. representativeness). As a result, 673 descriptors were retained for the next round of analysis.

##### The fifth round

Based on the results from the previous round, the interpreting scales team revised the descriptors further. Two workshops with over 20 trainers and professionals were then conducted to elicit more feedback. The participants of the workshops were asked to examine each descriptor, identifying well-written ones and commenting on problematic ones. For instance, the contents of some descriptors were contradictory (e.g. “*Can understand the source speech but cannot monitor the target language quality*”), while others were incomprehensible to lower-level students (e.g. “*Can deverbalize during interpreting*”). These descriptors were then reworded or discarded upon further discussion within the CSE-Interpreting team. Finally, 548 descriptors on interpreting competence remained as the first draft.

The draft scales were then circulated among experts, teachers, and interpreters to verify the appropriateness of the descriptors, their categorization, and levels. The draft was then revised into the first edition.

## Validating the Scale

The next step was to scale the descriptors through quantitative and qualitative validation. Validation of descriptors plays a key role in the construction and development of language ability scales (Brindley, 1998; North, 2000), especially for a national framework of language competencies with high stakes. Two rounds of validation were carried out in 2 years. First, large-scale surveys were used to collect data from interpreting trainers, students, and potential users of the scales around China. The data collected by questionnaires were analyzed using statistical methods, including Rasch modeling, to scale the descriptors and to test the representativeness of each descriptor and the appropriateness of the descriptor levels. Second, expert judgment and focus group interviews were implemented to corroborate and contextualize the findings from the quantitative analysis.

### Quantitative Validation: Rasch Scaling

A nationwide survey was first launched to collect quantitative data from potential users of the interpreting competence scales, including interpreting learners, trainers, and interpreters at various levels. This round of validation involved three steps:

#### Step 1: Questionnaire Design

The validation of interpreting competence descriptors was conducted within the context of the national project of CSE (Liu, 2019). In total, 5,046 descriptors from the eight CSE teams, including listening, speaking, reading, writing, and interpreting, were used to construct 80 sets of overlapping online/computerized adaptive questionnaires of 50 to70 items. Among them, 42 questionnaires contained the remaining 548 descriptors from the first draft of the CSE-Interpreting team. An excerpt from the sample questionnaire is provided in Supplementary Appendix 1.

As one of the aims of the Rasch methodology was to calibrate the descriptors onto a continuum, the questionnaires were linked though “anchor items” common to adjacent questionnaires. When covering a broad range of proficiency levels, this leads to an overlapping chain of questionnaires (targeted at successive levels), linked by the anchor items (North, 1995). Hambleton et al. (1991) recommended that anchor items comprise 20 to 25% of the total items. Following their suggestion, this project selected 20% of the total descriptors as anchor items. Using a similar approach to CEFR (North, 2000), we selected the anchor items from a larger pool of relevant items that were considered (by external experts and the author team) the most clear and representative.

In the questionnaire, each descriptor was followed by a rating scale from 0 to 4 with the following statements (translated from Chinese):

“0”: Cannot do it at all. (Unable to execute the task in any circumstances. Their proficiency is obviously much lower than this level.)
“1”: Can do it with significant help. (Can execute the task in favorable circumstances. Their proficiency is a bit lower than this level.)
“2”: Can do it. (Can execute the task independently in normal circumstances. Their proficiency is at this level.)
“3”: Can do it well. (Can execute the task even in difficult circumstances. Their proficiency is a bit higher than this level.)
“4”: Can do it easily. (Can execute the task easily in any conditions. Their proficiency is clearly much higher than this level.)

#### Step 2: Questionnaire Distribution and Data Collection

The participants for the nationwide survey of the CSE Project included 29,167 teachers and 120,710 students from around 300 primary schools, 600 high schools, and nearly 300 universities in 28 provinces, municipalities, and regions in China.

For the 42 questionnaires with descriptors of the interpreting scales, 5,787 teachers from 259 colleges and universities responded by rating the descriptors against their students’ actual Chinese–English interpreting performance, while 30,682 students from 215 colleges and universities took part by evaluating their own interpreting competence from June 20 to July 15, 2016. All teacher respondents were English teachers or T&I trainers. Most student respondents were English majors; only 3% of them were T&I majors. As the student population of T&I programs accounts for less than 5% of the large population of English language major students in China, 3% is considered sufficient.

To improve rating quality, the author team provided training to teachers (from the same institution) either in a half-day rating conference or via the CSE online working platform^1^. Several efforts were made to ensure the effectiveness of training. The half-day rating conference began with a brief presentation of the CSE Project and introduction of the rating procedures and was immediately followed by a mock rating session. During this session, teachers on site viewed video clips of three students’ oral English performance. Next, sample descriptors were simultaneously read by the conference host and shown on a large screen that could be seen by all participants. The teachers were asked to rate the students by raising number cards (0 to 4) based on the video clips and the descriptor. The host and the volunteers checked the rating results. As an example, during a conference in a university in Chongqing municipality (located in Southwest China), 58 English teachers took part in the mock rating session. For a sample descriptor, 45 teachers chose “3” or “4” (which was considered to be consistent with the student’s performance in the video), 11 teachers chose “1” or “2,” and two teachers chose “0.” The host invited the teachers who chose “1” or “0” to justify their ratings. The teachers who chose “0” said that they misunderstood “0” as “very easy.” The rating scales were explained once again to all teachers. The host explained to the teachers why “3” or “4” was closer to the students’ proficiency but also reminded them that there was no standard answer to the descriptors and that reaching consensus was not a requirement. The teachers were free to raise questions and discuss the rating results at any time. The teachers did not start answering the online questionnaire until all sample descriptors were evaluated in the mock session. Apart from the training on site, a mock rating session with further explanation was also provided in the CSE online working platform.

#### Step 3: Data analysis

The participants’ questionnaire responses were analyzed using FACETS 3.71.0 (Linacre, 2013). As Bond and Fox (2015) suggested, the Rasch rating scale model (RSM) can establish patterns in the rating scale categories in order to yield a single rating scale structure common to all the items on the scale. In this project, RSM analysis was performed by the CSE statistics team to estimate the relative difficulty of each of the interpreting competence descriptors, as rated by students and by teachers, and to examine the quality of rating scale responses. To determine how well the items measured the underlying traits and to examine the overall rating quality, we adopted a relaxed fit analysis cutoff of between 0.5 and 1.5 (Wright and Linacre, 1994) to determine overfit and misfit to the Rasch model.

Due to the large sample size, the standard error (SE) of the estimated parameters was 0.2 for the teachers’ evaluation and 0.08 for the students’ self-assessment. The Rasch difficulty parameters of the two data pools ranged from -0.5172 to 3.7848 for the teachers’ evaluation and from -0.9231 to 4.0254 for the students’ self-evaluation. In addition, 4.38% (*n* = 24) and 3.28% (*n* = 18) of the items from the teachers’ and students’ questionnaires, respectively, displayed both infit and outfit mean square values that were outside the cutoff range (0.5–1.5 logits). These items are considered psychometrically problematic; the misfitting examples are presented in Table 1.

**TABLE 1:** Examples of Misfitting Items From China Standards of English (CSE)-Interpreting Questionnaires.

Level	Descriptor	Difficulty and misfit estimates			
		Difficulty estimate (SE)	Outfit MNSQ (z-std)	Observed average	Expected average
	在口译任务前，能利用网络资源查询发言人的相关资料和背景。				
6	Can search for material and background information pertinent to the speaker using internet resources prior to interpreting.	−0.63(0.14)	1.82 (6.3)	2.75	2.82
	口译前，能借助互联网搜索相关话题的重要关键词。				
6	Can use the internet to search for keywords related to the topic of speech prior to interpr eting.	−0.78(0.15)	1.46 (3.8)	2.84	2.9
	能尊重并支持其他职业译员。				
6	Can respect and support other interpreters.	−1.16(0.15)	2.75 (9)	2.93	2.98
	能在口译中守时，并在无法守时的情况下，及时告知客户。				
6	Can be punctual for interpreting assignment and inform clients in a timely manner if the interpreter will be late.	0.12 (0.13)	2.14 (8.4)	2.53	2.58
	在无笔记交传中，能使用手头的工具，如手机查询生词或专业词汇。				
6	Can use hands-on tools, such as a mobile phone, to search for unfamiliar words or technical words during consecutive interpreting without notes.	−1.27(0.15)	1.81 (6.4)	2.97	3.01
	在口译时，能使用翻译辅助工具迅速查找口译中重要的生词。				
7	Can use computer-aided tools to search for important unfamiliar words during interpreting.	−0.99(0.19)	1.64 (4.2)	2.95	2.98
	在同声传译中，能在口译任务前检查相关设备，确保接收频道能接收发言人的声音，输出频道能传递自己的声音。				
8	Can inspect the relevant equipment to ensure the speaker’s voice can be received through the input channel, and the interpreter’s voice can be sent through the output channel prior to commencing simultaneous interpreting.	0.31 (0.19)	2.34 (6.5)	2.66	2.75
	在口译任务前，能联系主办方或讲话人获取会议资料等相关信息。				
8	Can contact event organizers or the speaker to collect pertinent information such as conference documents prior to interpreting.	−1.04(0.19)	1.93 (5.1)	2.94	2.97
	在口译任务前，能借助网络和词典等工具准备专业术语表。				
8	Can use tools such as the internet and dictionaries to create a glossary prior to interpreting.	−1.33(0.19)	1.84 (4.7)	3.02	3.05
	在同声传译中，能在听译的同时，利用网络资源查找相关术语。				
9	Can search for pertinent terminology using internet resources while listening and interpreting during simultaneous interpreting.	0.59 (0.2)	2.19 (6.2)	2.53	2.57

A second Rasch analysis was conducted after removal or revision of the misfitted items. This resulted in a second version of the CSE—Interpreting Competence Scales that included 304 items with both infit and outfit mean square values that fell between 0.5 and 1.5 logits.

The next analysis involved scaling. One way to check the acceptability and validity of the scale is to evaluate whether descriptors were calibrated in line with the original intentions of the design (North, 1995). To achieve that goal, appropriate cutoff points for scale levels need to be determined based on the logit scale. Setting pass/fail cutoff points requires precise conceptualization. There are many possible conceptualizations (North, 2000; Kolen and Brennan, 2004; Linacre, 2013). For this study, three factors were considered to locate the “zero” position in the scale: logit values were used in an attempt to create a scale with more or less equal intervals, patterns with natural gaps on the vertical scale, and a comparison of current patterns with levels in real life.

As illustrated in Table 2, there are nine levels in CSE. Level 5 is the center point, and each level covers approximately 0.7 logits. Similar to the results presented by North (1995), the range was slightly narrower in the middle of the scale and wider at the ends. As an integral part of the national project, the CSE-Interpreting team adopted the same cutoff range from Level 6 to Level 9. Reviewing the CSE-Interpreting data according to the cutoff points in Table 2, we found that 40% of the descriptors were consistent in terms of their actual and original difficulty levels. Meanwhile, 52% of the descriptors displayed a discrepancy of one level between their measured levels and provisional levels. Based on this, the misfitted descriptors and descriptors with significant discrepancies were modified based on the statistics.

**TABLE 2:** Scaling Results of CSE.

CSE level		Cutoff	Range on scale
Elementary	1	−2.39	
	2	−2.39 −1.65	0.74
	3	−1.65 −0.95	0.70
Intermediate	4	−0.95 −0.27	0.68
	5	−0.27 0.40	0.67
	6	0.40 1.08	0.68
Advanced	7	1.08 1.78	0.70
	8	1.78 2.52	0.74
	9	2.52	

The data from the quantitative validation also demonstrated that the overall difficulty of interpreting competence descriptors was comparatively higher than that of the English language proficiency descriptors (such as listening, speaking, reading, and writing). This result, once again, seems to differentiate interpreting competence from pure linguistic or bilingual competence. However, according to the data from the teachers’ and students’ evaluation, some descriptors that were deemed to be at Levels 6 and 7 before the scaling process were considered to be easier than the newly determined cutoff points of Level 6. This result implies that the difficulty of some interpreting competence descriptors was lower than Level 6. Therefore, the beginner’s level of CSE—Interpreting Competence Scales was revised to Level 5.

### Qualitative Validation: Revision

The second round of validation was designed to re-validate the descriptors, especially those that had been modified and re-adjusted previously. The qualitative methods used included survey and focus group interviews among English teachers, interpreting trainers, and interpreters.

#### Step 1: Survey Design

In total, 49 interpreting competence descriptors were selected for the second round of validation in questionnaires. Of these, 13 were revised descriptors, 21 were re-calibrated, 10 had been newly written by external experts after the quantitative validation, and 5 descriptors were those with significant discrepancies from their original levels according to the result of quantitative validation.

Together with other descriptors selected by the CSE teams, these 49 descriptors were compiled into 10 questionnaires. For Levels 4 to 6, they were embedded into questionnaires B1, B2, B3, B4, and B5. For Levels 7 to 9, they were embedded into questionnaires C1, C2, C3, C4, and C5. An excerpt of a sample questionnaire (C-3) is provided in Supplementary Appendix 2.

#### Step 2: Focus Group Interviews and Workshops

With the collective support of members of the CSE Project, 260 participants from various groups, including high school teachers, English teachers, and T&I trainers from universities, took part in 26 focus group interviews from six regions and provinces (Table 3) from March to July 2017. The interviews were designed to obtain feedback on the representativeness and appropriateness of the 49 descriptors collected by the CSE-Interpreting team.

**TABLE 3:** Questionnaire Allocation for Focus Groups.

Target population	Beijing	Guangdong	Hubei	Heilongjiang	Shandong	Yunnan
Grade 12 (high school)	B1	B5	B2	B3	B4	B5
EFL (English as a foreign language) course in Bachelor programs	B1		B2	B3	B4	B5
EFL course in Master’s programs	B1		B2	B3	B4	B5
Bachelor programs for English majors and BTI programs	C1		C2	C3	C4	C5
Master’s programs for English majors and MTI programs	C1		C2	C3	C4	C5

*BTI, Bachelor’s degree in Translation and Interpreting; MTI, Master’s degree in Translation and Interpreting.*

For each focus group interview, 10 English teachers of the targeted student population from at least three different schools or universities were recruited for a 3 h interview. One moderator led the interview with the help of two facilitators (all three were members of the CSE Project).

Before the focus group interview, written informed consent for participation was obtained. The use of the audio recorders was explained. Assurances of confidentiality and privacy in gathering, storing, and handling data were reiterated (Creswell, 2009), and participants were informed that they could withdraw from the interview at any time if they wished. In the interviews, the background and progress of this project was presented, and the purpose of the interview was explained in detail to the participants. The participants were also provided with an executive summary of the nine levels of CSE. Then, the teachers were divided randomly into smaller groups of three to four and worked for about 10 min to discuss and rate the sample descriptors with the guidance of the moderator in order to familiarize with the procedure. They were given the opportunity to ask any questions prior to the interview. The formal interview began with the moderator reading out each descriptor in the questionnaire. After 2 to 3 min of group discussion, the teachers were asked to show their scores by raising the number cards. They were then asked to explain their scoring and comment on the descriptors. They were asked to speak individually one at a time. If there were significant differences between the teachers’ scores, or discrepancies between teachers’ scores and provisional levels, the moderator could raise further questions. If any teacher had questions or comments about the description, he or she could discuss it briefly. It was unnecessary for the teachers to reach consensus. The moderator’s role was to ask questions and seek elaboration but stay neutral (Creswell, 2009). All 26 focus group sessions were simultaneously recorded in two ways: by a tape recorder, used with the permission of the participants, and by two facilitators who took notes during the session but did not participate in the discussion.

Besides the focus group interviews, the CSE-Interpreting team carried out two half-day workshops (4 to 5 h each) in July 2017. These workshops used all 49 descriptors and were conducted in the same format as shown in Supplementary Appendix 2. Four conference interpreting practitioners, who were also trainers, joined nine teachers of T&I programs (Table 4) in the workshops. While the same procedures were followed, in-depth discussion on the language and content of the descriptors was encouraged. Most participants were female and lecturers. About 55% of them were also practicing interpreters. Their professional background and teaching experience in T&I provided insights for descriptor refinement.

**TABLE 4:** Workshops With Interpreting Practitioners and Translation and Interpreting (T&I) Teachers.

Total number of participants		*N* = 13
Sex, *n* (%)	Female	9 (69)
	Male	4 (31)
Age, *years*	Mean	35.5
	Range	27–45
Position, *n* (%)	Professor	1 (8)
	Associate professor	2 (15)
	Lecturer	10 (77)
Highest level of education, *n* (%)	Master’s degree	7 (54)
	Doctorate degree	6 (46)
Interpreting experience (years as an interpreter), *n* (%)	1–4 years	3 (23)
	5–10 years	1 (8)
	11–15 years	3 (23)
	16 years and above	3 (23)
Teaching experience, *n* (%)	1–4 years	5 (38)
	5–10 years	3 (23)
	11–15 years	1 (8)
	16 years and above	4 (31)

#### Step 3: Data Analysis

The audio recordings of the interviews and workshops were transcribed with reference to the notes taken during the sessions. For instance, in Questionnaire C3, an English teacher from Heilongjiang province (located in Northeast China) commented on Descriptor No. 45 (“*Can identify the general idea of vague information according to the context during simultaneous interpreting for foreign affairs*”) as follows:

First of all, my students are unable to perform simultaneous interpreting even in the last semester of the program. Second, what do you mean by “vague information”? It seems to me that it is risky to explicate vague information in political settings, especially for diplomatic meetings.

The provisional level of this descriptor was Level 8 (MTI program and above), which is above the level of the students taught by the teacher. This result supported the appropriateness of the provisional level. The teacher’s second point reminded the research team to consider the representativeness and appropriateness of adjectives used in the descriptor.

For Descriptor No. 49 in Questionnaire B5 (“*Can accurately interpret daily conversation with normal speech during liaison interpreting*”), some English as a foreign language (EFL) teachers from universities in Yunnan province (in Southwest China) commented,

What does “liaison interpreting” mean? If I don’t understand this term, I am not sure if my students can do it. How do you define normal speed?

This comment indicates that “liaison interpreting” may be less familiar to some teachers and students. In terms of speech rate, the survey team explained the concept in detail (i.e. words per minute for slow, moderate, normal, and fast in the CSE—Interpreting Competence Scales).

In the workshops, most participants agreed with the level and content of the descriptors. Constructive suggestions were also offered to help refine the descriptors’ wording. For instance, “根据口译笔记(*according to the note-taking*)” should be changed to “借助口译笔记 (by note-taking)” for Descriptor No. 48 (“在外事接见的有笔记交传中，能根据口译笔记，译出当地国民生产总值、主要产业、未来发展方向等关键信息”); all the “翻译 (which could mean both ‘translation’ and ‘interpreting’)” should be changed to “口译 (interpreting)”; and “解决困难 (solve difficulties)” should be changed to “应对困难(overcome difficulties).”

Feedback from these verbatim transcripts was entered into Excel spreadsheets. Relevant metadata (e.g. questionnaire number, descriptor number, descriptor ID, category, provisional level) were also recorded. This feedback was then analyzed by both the CSE-Interpreting team and external experts to further revise the descriptors.

Results from both the focus groups and workshops showed that most of the participants agreed with the classification and descriptor levels. They felt that the descriptors were representative of typical interpreting activities. Most teachers, especially the interpreting trainers, agreed that interpreting competence descriptors were explicitly constructed and were generally easy to understand. Nevertheless, five types of problems associated with the 49 descriptors were identified and rectified through the qualitative validation, as shown in the following examples:

(1) Inconsistency. For example, “diplomatic interpreting” was phrased differently (“外事会见口译,” “外事接见口译,” and“外事会面口译”) in Chinese, despite that they all can refer to the same setting. In addition, “*search*,” “*collect*,” and “*look for*” were found in different levels of subscales of interpreting strategy. Although these words were used to refer to the same action, they may indicate different levels of difficulty. Therefore, revisions had to be made to ensure terminology consistency.
(2) Ambiguity. For example, “在新闻报道的有笔记交传中,能监控译语并在发生逻辑错误时及时与发言人确认并更正.” (*For consecutive interpreting using notes in a media setting, one can monitor their target language and confer with the speaker in a timely fashion when there is a logic error.*) In this descriptor, it is unclear whether the “*logic error*” is made by the speaker or the interpreter. The descriptor was revised by deleting the action initiator: “*For consecutive interpreting with notes in a media setting, one can monitor the logic error in their target language*.” In this way, the “*logic error*” could be made by either the speaker or the interpreter.
(3) Repetitiveness. Despite the five rounds of relevant analysis, some descriptors still seemed to be redundant. For instance, “在商务接待的无笔记交传中，能使用具体的表达形式，在译语表达中区分主要信息和次要信息.” (*For consecutive interpreting without notes in a business setting, one can use a specific expression to distinguish primary information and secondary information in the target language.*) Here, “具体的表达形式 (*specific expression*)” and “译语表达 (*target language*)” share the same meaning. The descriptor was then revised to “在商务接待的无笔记交传中，能在译语表达中区分主要信息和次要信息.” (*For consecutive interpreting without notes in a business setting, one can distinguish primary information and secondary information in target language*).
(4) Descriptors with similar meanings within the same level. For example, “能评估源语信息传递是否出现错误，包括重要信息、观点、细节和重要例子 (*Can evaluate whether there is an error in delivering the source information, such as key information, opinions, details and important examples*)” and “能评估核心信息遗漏、逻辑结构混乱、关键术语误译等重大错误 (*Can evaluate major errors such as core information loss, confusing logical structure, key terms mistranslation*)” were both found in the strategy subscale of Level 8. These two descriptors essentially touched on similar abilities. In this case, we revised the first descriptor into “在同声传译中，我能评估并修正源语信息传递中出现的错误 (*In simultaneous interpreting, I can evaluate and correct major errors*),” by adding the specific interpreting mode to differentiate it from interpreting in general.
(5) Untypical activities. For example, in the descriptors “在商务接待的无笔记交传中，能译出原料价格信息之间的逻辑关系 (*For consecutive interpreting without notes in business receptions, one can identify the logical relationship between raw material prices*),” participants in the interviews felt that “raw material prices” were seldom mentioned in the scenarios of “business reception.” Therefore, the descriptor was revised as “在商务接待的无笔记交传中，能概括地译出接待方行程安排等信息 (*For consecutive interpreting without notes in business receptions, one can interpret the itinerary and other information briefly*).”

Based on the results of the quantitative and qualitative validation, an external expert group consisting of researchers, trainers, and interpreters were invited to refine the descriptors. Eleven descriptors with typical interpreting activities were newly written by these experts. Upon the request of the CSE Project, the author team wrote descriptors to summarize the interpreting performance of each level. Sixteen descriptors were written for the Overall Interpreting Performance Scale, and 28 were written for the Self-assessment Scale for Interpreting Competence.

Upon final refinement by the Chinese editors, 12 scales with 369 descriptors and five levels were developed for interpreting competence: Overall Interpreting Performance (1 scale, 16 descriptors), Interpreting Competence in Typical Interpreting Activities (6 scales, 220 descriptors), Interpreting Strategy (4 scales, 105 descriptors), and Self-assessment for Interpreting Competence (1 scale, 28 descriptors). Supplementary Appendix 3 provides two examples of CSE—Interpreting Competence Scales in English, and the full English version can be accessed on the National Education Examinations Authority (NEEA) website (see text footnote 1).

## Discussion

### Description of Cognitive Ability

Cognitive ability is regarded as a core element of the interpreting competence construct. However, it is not feasible to operationalize it in the description stage. Based on several meetings and discussions among the project team members, the features of interpreting’s cognitive process have been conceptualized in different settings. Cognitive activities are described through the process and the product of interpreting, such as identifying, retrieving, summarizing, analyzing, anticipating, and monitoring.

The description of cognitive ability sometimes appears to overlap with typical interpreting activities. For example, one may find descriptors with similar cognitive abilities in the subscales of Typical Interpreting Activities and scales for Overall Interpreting Performance. The two sets of scales differ because the first focuses on a few real-life interpreting settings and is of practical use in the workplace, while the second pertains to the core part of interpreting competence at each level.

### Description of Interpreting Strategies

Interpreting strategies have, in some cases, turned out to be an unfamiliar or confusing concept for some teachers and students. This confusion may be related to how interpreting is taught and studied in the Chinese context. Compared with the product of interpreting (i.e. performance), the process of interpreting can easily be overlooked in interpreting training and learning. There has been very sparse coverage and minimal guidance in relevant training syllabuses on cognitive task analysis in interpreting, let alone the teaching of interpreting strategies (since most of the interpreting courses in China are skills-oriented). Through rigorous training and detailed illustration during the exemplar-generation stage, the teachers may consider demonstrating some useful strategies often used by their students in the classroom or in their after-class interpreting exercises. When dealing with strategy descriptors, we grouped abstract descriptors concerning planning, monitoring, and evaluating into metacognitive strategies; meantime, we categorized specific and concrete descriptors involving inferencing, elaborating, summarizing, repeating, and note-taking into cognitive strategies. However, descriptors related to emotion or social interaction that might be categorized as socio-affective strategies (Vandergrift, 1997) were not yet included.

### Interpreting Task Difficulty

As the scales are designed to be applied in the Chinese educational system, the descriptors are required to be explicit and internally consistent. For the interpreting competence descriptors, we usually used criteria such as delivery speed, length of the speech, and topic and lexical complexity to indicate the difficulty level of interpreting materials. However, in the case of delivery speed, what exactly is the difference between “delivered slowly” and “delivered at normal speed”? In our study, we distinguished between four levels of delivery speed—slow, moderate, normal, and fast—and then defined each level:

*“Fast (in English): approximately 140–180 words/min; moderate speed (in English): approximately 100–140 words/min; fast (in Chinese): approximately 160–220 Chinese characters/min; moderate speed (in Chinese): approximately 120–160 Chinese characters/min”* (National Education Examinations Authority, 2018).

This level-defining approach could also be applied to other criteria, although decisions should be made carefully based on rigorous theoretical underpinning and sufficient evidential support. Similar to the validation of CEFR and other related scales, we should continue to collect relevant data in order to fine-tune interpreting competence descriptors.

### Interpreting Modes and Levels in the Scales

**FIGURE 4:**
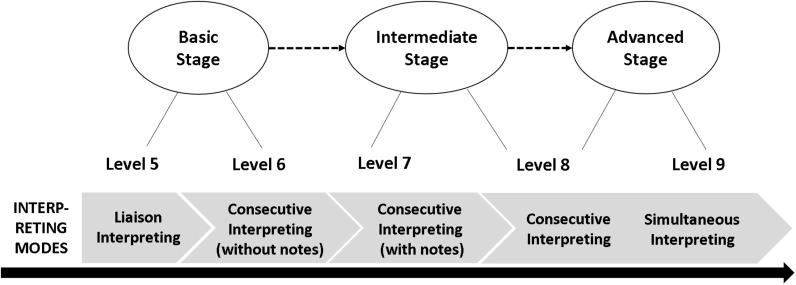
Interpreting modes vs. levels in the Scales.

To indicate the developmental stages of interpreting competence, five levels are used to represent the three classic stages of basic, intermediate, and advanced competence (Figure 4). For instance, Levels 5 and 6 are basic stages, at which one can complete liaison interpreting tasks. Typical interpreting activities at these levels are relatively simple and informal, with moderately slow speech rate and short segments. These could include a guided tour, guest reception, informal visit, or business escort. Levels 7 and 8 are the intermediate stages; student interpreters at these levels should be able to complete interpreting tasks with longer segments and in more formal settings. In particular, Level 8 involves the advanced stage of consecutive interpreting and the introductory stage of simultaneous interpreting. In other words, Level 8 represents the transition from beginner to advanced learners. Level 9 represents the most difficult tasks and the almost “perfect” performance of interpreting.

Although the scales were developed in a mixed-methods empirical approach, establishing the cutoff points between levels was, in part, a subjective procedure. While some students, teachers, researchers, or institutions may prefer broad levels, others prefer finer levels. The advantage of this three-stage-branching approach is that “a common set of levels and/or descriptors can be ‘cut’ into practical local levels at different points by different users to suit local needs and yet still relate back to a common system” (Council of Europe, 2001). According to specific purposes (teaching, learning, and testing, for example), users of the scale can introduce sub-levels to the scales to fit their specific needs.

## Limitations and Applications

Despite the tremendous efforts involved in the CSE Project, there are still a number of limitations. First, because of the logistical constraints, only 2.54% of the participants in this study were T&I teachers or students. As the scales were designed for a wide range of potential T&I teachers, students, and staff working in corporations and government agencies, they warrant further revision and validation to ensure that they appropriately reflect professional practice. Second, although the Rasch-based results were quite encouraging, the relevant analyses were conducted collectively by the statistics team of the CSE Project based on the data of descriptors from all CSE teams. As a result, we did not obtain the Wright maps describing our data on interpreting competence. Finally, the 55 newly written descriptors created by the external experts and the author team after the qualitative validation need to be validated based on the steps described in Section 3, *Validating the scale*.

In terms of application, the Interpreting Competence Scales can be operationalized for teaching, learning, and assessment purposes. First, interpreting trainers can make use of the levels and corresponding descriptors of the scale in their teaching plans, pedagogy, and teaching materials. For these applications, it will be useful to transform the descriptors into classroom tasks at different stages of interpreting training. This allows trainers to use descriptors to evaluate performance, develop teaching materials, and examine the appropriateness of the descriptors. Second, although students’ self-study contributes to the development of interpreting competence, little guidance is available for material selection and performance assessment (Wang, 2015). The Self-Assessment Scale for Interpreting Competence can be used by students to self-diagnose and evaluate their learning outcomes. It potentially provides students with opportunities to understand their current level of interpreting competence, assess their performance, and set specific goals for further improvement. Further research is required to investigate the effectiveness and washback of the Self-Assessment Scale in students’ self-directed practice.

Third, testing and assessment of interpreting competence is an important area in which our scales are expected to play a major role. The scales offer a window into interpretation aptitude by setting levels of baseline competence. They also provide detailed information about interpreting activities, strategies, and requirements for interpreting quality at different levels. Given the detailed descriptors, teachers and testers may be able to use the scales to inform the development of aptitude tests, diagnostic tests, and formative and summative assessments of interpreting. In addition, existing tests (e.g. NAATI and CATTI) should be aligned to the standardized descriptor scales. The focus of alignment should be on characteristics of practice domains (e.g. subject matter, interpreting activities), difficulty levels, and the rating methods. Such alignment would help achieve greater consistency and coherence in interpreting education and facilitate communication among interpreting trainers, learners, test developers, professionals, and policymakers.

## Data Availability Statement

The data sets generated for this study are available on request to the corresponding author.

## Ethics Statement

Ethical review and approval was not required for the study on human participants in accordance with the local legislation and institutional requirements. The participants provided their written informed consent to participate in this study.

## Author Contributions

WW and YX wrote the initial draft of the manuscript. BW advised on the structure and revised the manuscript substantially. LM led the relevant project on the development of scales for interpreting competence.

## Conflict of Interest

The authors declare that the research was conducted in the absence of any commercial or financial relationships that could be construed as a potential conflict of interest.

## Footnotes

1 http://cse.neea.edu.cn
