# Supplementary material for: Developing Interpreting Competence Scales in China
Source: Front Psychol. 2020 Apr 23;11:481. doi: 10.3389/fpsyg.2020.00481 (PMC7197373; doi:10.3389/fpsyg.2020.00481)
Supplement: Supplementary file 1 [file Data_Sheet_1.docx]

**Appendix 1:**

**Sample questionnaire for teachers L8-1**

**(Translated version)**

**∽∽∽∽∽∽∽∽∽∽∽∽∽∽∽∽∽∽∽∽∽∽∽∽∽∽∽∽∽∽∽∽**

**The teacher:**

1. Teacher’s name: .
2. Sex: □ M □ F
3. Age: □ under 25 □ 25-30 □ 31-35 □ 36-40 □ 41-45 □ 46-50
4. Years of teaching: □ 1-4 □ 5-8 □ 9-12 □ above 12
5. Your education: □ Specialist degree or below □ Bachelor‘s degree □ Master’s degree □ Ph.D.
6. Your students’ level (please indicate the grade of students you are teaching currently): .
7. Email address: .
8. Province: .
9. Name of school: .

Please select a medium-level learner in your class and rate him/her for the following descriptors of English language proficiency.

**The learner:**

Name: .

Grade/Level of study: .

Major (for college student or above): .

Sex: □ M □ F

Please rate the learner for each item on the questionnaire using the following scale. Please select the appropriate number next to each item.

| **0** | **1** | **2** | **3** | **4** |
| --- | --- | --- | --- | --- |
| **Cannot do it at all** | **Can do it with much help** | **Can do it** | **Can do it well** | **Can do it easily** |
| Unable to execute the task in any circumstances. His/her proficiency is obviously much lower than this level. | Can execute the task in favorable circumstances. His/her proficiency is a bit lower than this level. | Can execute the task independently in normal circumstances. His/her proficiency is at this level. | Can execute the task even in difficult circumstances. His/her proficiency is a bit higher than this level. | Can execute the task easily in any conditions. His/her proficiency is clearly much higher than this level. |

**Interpreting**

| 1 | 在外事接见的有笔记交传中，能按照译语习惯，准确表达源语的信息、话语风格和语域。  Can follow target-language norms to reflect source-language register and style during CI with notes for a foreign affairs meeting. | 0 | 1 | 2 | 3 | 4 |
| --- | --- | --- | --- | --- | --- | --- |
| 2 | 在外事接见的无笔记交传中，能即时对源语中的琐碎、冗余信息进行概述。  Can identify and deal appropriately with redundant information during CI with notes for a foreign affairs meeting. | 0 | 1 | 2 | 3 | 4 |
| 3 | 在有笔记交传中，能迅速切分话语的意义单位并进行有选择的记忆。  Can chunk source-language units of meaning and memorize information selectively during consecutive interpreting. | 0 | 1 | 2 | 3 | 4 |
| 4 | 在同声传译中，能利用语流缩短译语与源语发言之间的时间差。  Can adjust ear-to-voice span (EVS) based on the speech flow during simultaneous interpreting. | 0 | 1 | 2 | 3 | 4 |
| 5 | 能根据知识和逻辑，判断讲话者的表达错误并在翻译中予以纠正。  Can use pre-existing knowledge and contextual logic to determine and correct obvious errors committed by the speaker. | 0 | 1 | 2 | 3 | 4 |
| 6 | 在口译中遇到特殊术语时，能询问讲话者或者请教现场的相关专家以确保信息准确。  Can solicit assistance from the speaker or nearby experts to ensure accuracy whenever unusual terminology is encountered. | 0 | 1 | 2 | 3 | 4 |
| 7 | 在商务洽谈的会议同传中，能理解讲话中专业性较强的信息。  Can comprehend specialized information presented by the speaker during simultaneous interpreting with notes for business negotiations | 0 | 1 | 2 | 3 | 4 |
| 8 | 在口译任务前，能了解发言相关专题的知识。  Can acquire the information and resources relevant to the interpreting assignment prior to interpreting. | 0 | 1 | 2 | 3 | 4 |
| 9 | 在同声传译中，能将源语的句子以意群或概念为单位恰当切分并译出。  Can chunk source-language information to complete target-language speech during simultaneous interpreting. | 0 | 1 | 2 | 3 | 4 |
| 10 | 在同声传译中，能在预测信息的同时，监控译语是否流畅。  Can anticipate information while at the same time monitoring the fluency of the target-language information during simultaneous interpreting. | 0 | 1 | 2 | 3 | 4 |
| 11 | 在商务洽谈的无笔记交传中，能分析源语信息间的逻辑关系。  Can analyze the logical relationship that link key information in the negotiations during CI with notes for business negotiations. | 0 | 1 | 2 | 3 | 4 |
| 12 | 在科普讲座的同声传译时，能理解源语中的专业性较强的信息。  Can understand technical information present in the source-language speech during consecutive interpreting with notes for popular science lectures. |  |  |  |  |  |
| 13 | 在文化交流的同声传译中，能在传达源语信息之外，传达其强调的内容和语气。  Can accurately deliver speech emphasis, such as tone during consecutive interpreting with notes for cultural communication. | 0 | 1 | 2 | 3 | 4 |
| 14 | 在艺术沙龙的有笔记交传中，能用符合译语文化习惯的方式重组源语信息。  Can reorganize source-language information to correspond with target-language cultural norms during consecutive interpreting with notes for an art salon. | 0 | 1 | 2 | 3 | 4 |
| 15 | 在学术报告的同声传译中，能恰当增减源语中的次要信息。  Can add or delete source-language secondary information during simultaneous interpreting for an academic talk. | 0 | 1 | 2 | 3 | 4 |
