# Supplementary material for: Developing Interpreting Competence Scales in China
Source: Front Psychol. 2020 Apr 23;11:481. doi: 10.3389/fpsyg.2020.00481 (PMC7197373; doi:10.3389/fpsyg.2020.00481)
Supplement: Supplementary file 2 [file Data_Sheet_2.docx]

**Appendix 2:**

**Sample Questionnaire (No.C3) for Focus Group**

**(Translated version)**

**∽∽∽∽∽∽∽∽∽∽∽∽∽∽∽∽∽∽∽∽∽∽∽∽∽∽∽∽∽∽∽∽**

**The teacher:**

1. Teacher’s name: .
2. Sex: □ M □ F
3. Age: □ under 25 □ 25-30 □ 31-35 □ 36-40 □ 41-45 □ 46-50
4. Years of teaching: □ 1-4 □ 5-8 □ 9-12 □ above 12
5. Your education: □ Specialist degree or below □ Bachelor‘s degree □ Master’s degree □ Ph.D.
6. Your students’ level (please indicate the grade of students you are teaching currently): .
7. Email address: .
8. Province: .
9. Name of school: .

Please select a medium-level learner in your class and rate him/her for the following descriptors of English language proficiency.

**The learner:**

Name: .

Grade/Level of study: .

Major (for college student or above): .

Sex: □ M □ F

Please rate the learner for each item on the questionnaire using the following scale. Please select the appropriate number next to each item.

| **0** | **1** | **2** | **3** | **4** |
| --- | --- | --- | --- | --- |
| **Cannot do it at all** | **Can do it with much help** | **Can do it** | **Can do it well** | **Can do it easily** |
| Unable to execute the task in any circumstances. His/her proficiency is obviously much lower than this level. | Can execute the task in favorable circumstances. His/her proficiency is a bit lower than this level. | Can execute the task independently in normal circumstances. His/her proficiency is at this level. | Can execute the task even in difficult circumstances. His/her proficiency is a bit higher than this level. | Can execute the task easily in any conditions. His/her proficiency is clearly much higher than this level. |

1. 在外事会谈的同声传译中，能根据上下文辨析模糊信息的大意。

Can identify the general idea of vague information according to the context during simultaneous interpreting for foreign affairs.

| 该学生：  The student: | 您作此判断的依据是：  Your judgment is based on: |
| --- | --- |
| □ 0. 完全做不到  Cannot do it at all | □ A. 根据教学中的观察  Observation in teaching |
| □ 1. 勉强做到  Can do it with much help | □ B. 根据对学生的了解推测  My understanding of the students’ proficiency. |
| □ 2. 基本做到  Can do it | □ C. 描述语难以理解，无法判断  The descriptor is difficult to understand and I cannot make a judgement. |
| □ 3. 较好做到  Can do it well | □ D. 其他  Other reasons. |
| □ 4. 完全做到  Can do it easily |  |

1. 在有笔记交传中，能借助笔记，准确译出源语的主要信息。

Can accurately interpreting the main idea of the source speech with the help of note-taking during CI.

| 该学生：  The student: | 您作此判断的依据是：  Your judgment is based on: |
| --- | --- |
| □ 0. 完全做不到  Cannot do it at all | □ A. 根据教学中的观察  Observation in teaching |
| □ 1. 勉强做到  Can do it with much help | □ B. 根据对学生的了解推测  My understanding of the students’ proficiency. |
| □ 2. 基本做到  Can do it | □ C. 描述语难以理解，无法判断  The descriptor is difficult to understand and I cannot make a judgement. |
| □ 3. 较好做到  Can do it well | □ D. 其他  Other reasons. |
| □ 4. 完全做到  Can do it easily |  |
