# Supplementary material for: Developing Interpreting Competence Scales in China
Source: Front Psychol. 2020 Apr 23;11:481. doi: 10.3389/fpsyg.2020.00481 (PMC7197373; doi:10.3389/fpsyg.2020.00481)
Supplement: Supplementary file 3 [file Data_Sheet_3.docx]

**Appendix 3: Examples of the CSE-Interpreting Scales**

**Example (1) Overall interpreting ability Scale**

As the interpreting ability is based upon certain bilingual competence, the overall scale for interpreting ability starts from Level 5.

| CSE 9 | - Can accurately, completely, and fluently interpret speech on subjects of a wide range of categories, including technical speech relating to unfamiliar fields. - Can interpret in high-level, formal, and informal speech contexts, ensuring the register and style of target language speech is consistent with that of the source language. - Can use and integrate consecutive or simultaneous interpreting skills and strategies, and deliver adaptable, idiomatic language. |
| --- | --- |
| CSE 8 | (Consecutive Interpreting)   - Can interpret information-dense speech (e.g. foreign affairs meetings, media reports) in which segments are comparatively long and delivered at a relatively high speech rate with the aid of note-taking or other methods. - Can interpret specialised discourse (e.g. academic lectures, court hearings) fluently and professionally. - Can make timely adjustments to target-language speech based on on-site circumstances in order to ensure accurate and complete delivery, keeping as close as possible to source-language register and style.   (Simultaneous Interpreting)   - Can interpret moderate information-dense speech (e.g. as in news conferences, industry forums) delivered at a regular speed with little to no accent interference. - Can coordinate efforts in simultaneous interpreting for anticipation, chunking, decalage, and adjustment, and ensure relatively accurate, complete, fluent, and idiomatic delivery. |
| CSE 7 | - Can consecutively interpret moderate information-dense speech (e.g. as in business negotiations, training activities) in which segments are comparatively short and delivered at a regular speed with note-taking notes. - Can use methods such as addition, deletion, and explication to interpret important source-language information and key details, ensuring logical, coherent, appropriate, and fluent delivery. - Can promptly discover interpreting errors such as misinterpretation or omission of important information; and take immediate actions during follow-up interpreting. |
| CSE 6 | - Can consecutively interpret comparatively short speech on a prepared topic (e.g. as in everyday interactions with visitors, trade fairs) without taking notes. - Can actively anticipate speech information, monitor target-language accuracy and completeness, and correct mistakes. |
| CSE 5 | - Can perform liaison interpreting on a familiar topic (e.g. greetings and goodbyes, escorted shopping assignments). - Can interpret important source-language information based on communicative settings and contextual knowledge, ensuring overall accuracy of meaning. - Can be aware of and try to correct obvious errors that occur during interpreting. |

**Note:** the “speed” mentioned in this Table and Table 85 is defined as follows:

Fast (in English): approximately 140-180 words/min;

Moderate speed (in English): approximately 100-140 words/min;

Fast (in Chinese): approximately 160-220 Chinese Characters/min;

Moderate speed (in Chinese): approximately 120-160 Chinese Characters/min.

**Example (2) Self-assessment scale for interpreting ability**

| CSE 9 | - Prior to interpreting, I can familiarise myself with the speaker’s speech habits, such as pronunciation and intonation, rate of speech, and wording preference. - During difficult, specialised consecutive interpreting, such as a heads-of-government meeting or a court hearing for an important case, I can accurately, completely, and fluently interpret source-language information while ensuring target-language delivery is consistent with the speaker’s register and style. - During consecutive interpreting, I can use and integrate consecutive interpreting skills and strategies to deliver adaptable, idiomatic language, with a natural and relaxed bearing and clear enunciation. - During simultaneous interpreting for a government press conference or report of a major emergency, I can interpret source-language information accurately, completely, and fluently. - During simultaneous interpreting, I can use and integrate simultaneous interpreting skills and strategies to deliver appropriate, adaptable, idiomatic language with clear enunciation and a pleasant voice. - During interpreting, I can identify and handle appropriately any significant errors made by the speaker. |
| --- | --- |
| CSE 8 | - Prior to interpreting, I can familiarise myself with industry-related specialised vocabulary, background information, and development trends. - During consecutive interpreting in which speech segments are comparatively long, such as news conferences, academic talks, or business negotiations, I can draw support from my notes to interpret information-dense, relatively specialised speech that is delivered at a regular speed with a certain degree of accent. - During consecutive interpreting in which speech segments are comparatively long, I can use context and related background knowledge to analyse the speaker’s logic. - During consecutive interpreting in which speech segments are comparatively long, I can use devices such as addition, omission, explication, and word reordering to ensure the target language is produced accurately, completely, and fluently. - I can avoid committing obvious grammar mistakes and deliver the target language fluently. I can also use non-verbal methods to ensure effective communication. - During simultaneous interpreting, such as for a political leader’s speech, a live-broadcast of a sporting competition, or an expert review meeting, I can interpret low-information-density speech delivered at a regular speed and without any obvious grammar mistakes. - During simultaneous interpreting, I can use strategies such as anticipation, omission, and summarisation to divide my attention. I can simultaneously listen and analyse information, using suitable sense groups to chunk source-language sentences to interpret the speaker’s message relatively accurately, completely, and fluently. - During interpreting, I can monitor in real-time the accuracy, fluency, and cohesion of target-language information. - I can assess and correct errors that occur in the delivery of source-language information. |
| CSE 7 | - I can draw support from my notes to interpret moderately information-dense speech in which speech segments are comparatively short and delivered at a regular speed, as seen in activities such as business visits, popular science lectures, or guided tours. - Prior to interpreting, I can use information about the subject and the speaker’s background to create a glossary, gain pertinent knowledge, and actively anticipate source-language information. - During interpreting, I can understand, analyse, and remember, using my notes and memory, the speaker’s entire message. - I can use relatively fluent target language to deliver source-language information. - I can use materials prepared prior to interpreting or my accumulated experience to help when I encounter difficulties. |
| CSE 6 | - I can perform consecutive interpreting without note-taking for a familiar topic in which speech segments are comparatively short, such as when receiving business people or accompanying people on a tour. - I can use a variety of channels to make preparations, such as contacting event organisers or using the internet, to collect material pertinent to an interpreting assignment and a speaker’s background. - I can understand the speaker’s main intent, remember principal information of a speech, and use the target language to deliver source-language information relatively accurately. - I can monitor target-language accuracy and completeness and promptly correct mistakes. - I can request help from the speaker or the audience when I encounter difficulties. - After the interpreting assignment, I can reflect on my performance and on the reasons for any difficulties I encountered. |
| CSE 5 | - I can integrate subject and pertinent background knowledge to interpret important information present in a dialogue while interpreting for a familiar topic, such as airport pick-up and drop-off or a shopping assignment. - Prior to interpreting, I can make pertinent preparations, such as familiarising myself with an itinerary, subject, and details of an activity. - I can recognise and promptly correct obvious errors that occur during interpreting. |
